# The prevalence of mental disorders in adults in different level general medical facilities in Kenya: a cross-sectional study

**DOI:** 10.1186/1744-859X-8-1

**Published:** 2009-01-14

**Authors:** David M Ndetei, Lincoln I Khasakhala, Mary W Kuria, Victoria N Mutiso, Francisca A Ongecha-Owuor, Donald A Kokonya

**Affiliations:** 1 Department of Psychiatry, University of Nairobi, Nairobi, Kenya; 2 Africa Mental Health Foundation (AMHF), P.O. Box 48423, 00100-GPO, Nairobi, Kenya; 3 Coast Provincial General Hospital, Mombasa, Kenya; 4 Kakamega Provincial General Hospital, Kakamega, Kenya

## Abstract

**Background:**

The possibility that a significant proportion of the patients attending a general health facility may have a mental disorder means that psychiatric conditions must be recognised and managed appropriately. This study sought to determine the prevalence of common psychiatric disorders in adult (aged 18 years and over) inpatients and outpatients seen in public, private and faith-based general hospitals, health centres and specialised clinics and units of general hospitals.

**Methods:**

This was a descriptive cross-sectional study conducted in 10 health facilities. All the patients in psychiatric wards and clinics were excluded. Stratified and systematic sampling methods were used. Informed consent was obtained from all study participants. Data were collected over a 4-week period in November 2005 using various psychiatric instruments for adults. Descriptive statistics were generated using SPSS V. 11.5.

**Results:**

A total of 2,770 male and female inpatients and outpatients participated in the study. In all, 42% of the subjects had symptoms of mild and severe depression. Only 114 (4.1%) subjects had a file or working diagnosis of a psychiatric condition, which included bipolar mood disorder, schizophrenia, psychosis and depression.

**Conclusion:**

The 4.1% clinician detection rate for mental disorders means that most psychiatric disorders in general medical facilities remain undiagnosed and thus, unmanaged. This calls for improved diagnostic practices in general medical facilities in Kenya and in other similar countries.

## Background

Mental disorders are more common in medical than in community settings [1], and some studies report that up to 40% of the patients in general medical and surgical wards are depressed and require treatment [2,3]. This level exceeds the 20 to 25% prevalence rates reported in studies carried out in general outpatient facilities in Kenya [4,5]. The most frequent diagnoses of mental illnesses made in general hospital settings are depression, substance abuse, neurotic stress-related and anxiety disorders, [6] and these are more frequently associated with chronic medical conditions [7-9]. However, since most patients present at health facilities with medical rather than psychiatric complaints, these diagnoses may be missed especially if the levels of somatic symptoms are elevated [10]. This is especially so considering that some chronic medical illnesses and psychiatric disorders may produce similar somatic symptoms [11]. Conversely, almost 60% of psychiatric patients have identifiable physical illnesses [12].

Untreated psychiatric illness is associated with increased morbidity, a longer hospital stay and ultimately, increased costs of care [13]. This often leads to wasteful, costly and inefficient use of medical services and complications of the diagnoses and treatments among these patients [14]. Therefore, early detection and treatment of mental disorders, which in most cases is the responsibility of non-psychiatric medical personnel, is essential, especially since symptoms of mental disorders are frequently not recognised.

The possibility that a significant proportion of the patients attending a general health facility may have a mental disorder means that psychiatric conditions must be recognised and managed appropriately. However, in Kenya, there are only 68 psychiatrists serving a population of approximately 34 million. Less than half of them are involved in active clinical work, and they mainly practice in the major urban areas meaning that rural populations remain grossly underserved with the result that for the majority of patients, psychiatric disorders remain untreated. With no data on prevalence and detection rates of psychiatric disorders in Kenyan hospitals, it is not possible to convince policy makers to assign mental health personnel as an integral part of the professional body in general hospitals. Such a move will facilitate the training of non-psychiatric staff, especially those at primary health care levels, on how to recognise, manage and make appropriate referrals for patients since it is unlikely that, in Kenya, enough psychiatrists will be trained in the foreseeable future [15]. This study therefore aimed to document the prevalence and detection of mental health problems across all levels of general medical facilities, from the primary health care level to the national level.

## Methods

**Figure 1:**
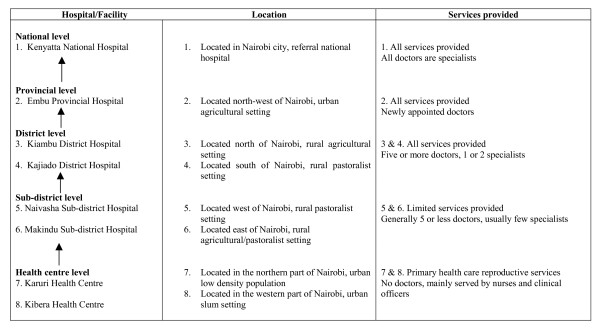
**The referral structure of public medical facilities in Kenya**. Two private health facilities were also included in the study. Magadi hospital is located in a rural pastoralist setting, north of Nairobi, and Kikuyu hospital located west of Nairobi is found in a predominantly agricultural rural setting. Both are served by privately employed doctors and provide elementary health services.

This was a cross-sectional survey conducted in 10 health facilities that were selected to represent all levels of health provision (from primary health care centre to the national level), different economic environments within which the facilities are located (industrial, agricultural, nomadism) as well as the different training levels of medical personnel. The health facilities to represent the above spectrum were selected on the basis of their proximity (within a 200 km radius) to Nairobi, the capital city of Kenya. The different health care levels in Kenya and a brief description of the facilities studied are summarised in Figure 1.

Two health centres (Karuri and Kibera), two subdistrict hospitals (Makindu and Naivasha), two district hospitals (Kiambu and Kajiado), one provincial hospital (Embu) and one national teaching and referral hospital (Kenyatta National Hospital (KNH)) were selected. Also included were one faith-based hospital (Kikuyu) and one private institutional hospital (Magadi). All the facilities except for health centres offer both inpatient and outpatient services.

Using a list of all health facilities within the radius of the study, a broad stratified sampling method was applied in order to first select facilities representing each level of health care provision and then those representing different medical specialties in each facility. In each area of specialty, a systematic sampling method was employed until the required number of patients was achieved. The purpose of the study was explained to the patients and instructions on how to complete the self-administered instruments were provided. All inpatients and outpatients who were not too sick to participate and those who were able to comprehend the instructions, complete the questionnaires and to provide informed consent for voluntary participation were recruited into the study. No patients were recruited from the psychiatric units of any of the health facilities visited and no maternity cases were included.

The data were collected over a 4-week period in November 2005. A questionnaire was verbally administered on all the patients to elicit information on their sociodemographic profiles. The following instruments, which are recognised as having good psychometric properties, were also administered to obtain information on psychiatric disorders: Beck Depression Inventory (BDI) [16], the Leeds Scale for the Self-Assessment of Anxiety and Depression (LSAD) [17], the Ndetei-Othieno-Kathuku Scale (NOK) [18,19], the Mini-Mental State Examination (MMSE) [20] and the Composite International Diagnostic Interview (CIDI) screen for psychosis [21]. Descriptive data were generated using SPSS V, 11.5 (SPSS, Chigaco, IL, USA) and these were analysed to determine underlying patterns. The results are presented in narrative form and in tables.

## Results

A total of 2,770 patients aged 18 years and older were recruited into the study. There were varied response rates for all the variables across all the sites. KNH had the highest proportion of patients (65%, n = 1,801) and Kibera health centre had the lowest (1.2%, n = 33). Figure 1 shows the referral structure of public medical facilities in Kenya.

### Sociodemographic characteristics

As shown in Table 1, the ages of the patients ranged from 18 to 92 years (mean age = 34.2 years) and more than half of the patients (52.4%) were aged 30 years or less. Overall, 46.3% of the patients were male. The patients were predominantly Christian (94.9%, 2,555/2,692) and 3.8% (n = 108) were Muslims. More than one-third (34.8%, 938/2,696) of the patients had never been married. Of those who were married, 38 (1.4%) were in polygamous unions and the highest rates of polygamy were recorded in Kajiado.

**Table 1:** Sociodemographic characteristics (%)

**Variables**	**All sites^a^**	**KNH**	**Embu**	**Kiambu**	**Kikuyu**	**Kajiado**	**Kibera**	**Makindu**	**Naivasha**	**Magadi**	**Karuri**
**Age (years)**	**2,770**	**1,801**	**177**	**161**	**200**	**61**	**33**	**123**	**89**	**82**	**43**
18 to 30	52.4	49.6	55.3	56.8	59.0	52.5	70.50	53.00	61.60	68.00	74.4
31 to 45	28.6	29.1	33.7	26.5	27.5	18.1	26.40	29.10	49.20	23.20	18.5
46 to 60	13.9	16.0	7.3	10.0	10.0	21.8	2.90	11.20	11.00	5.60	6.9
61 to 75+	5.1	5.3	4.7	6.9	4.0	8.1	0	38.0	3.3	0	0
**Sex**	**2,759**	**1,795**	**175**	**161**	**200**	**61**	**33**	**123**	**86**	**82**	**43**
Male	46.3	44.7	43.4	65.4	48.4	66.7	51.5	37.3	30.2	46.5	50
Female	53.7	55.3	56.6	34.6	51.6	33.3	48.5	62.7	69.8	53.5	50
**Religion**	**2,692**	**1,753**	**170**	**157**	**198**	**57**	**30**	**119**	**84**	**81**	**43**
Christian	94.9	96.2	100.0	89.9	99.0	73.7	74.2	88.5	96.5	89.7	100.0
Others	5.0	3.8	0	10.1	1.0	24.0	25.8	11.5	3.3	10.4	0
**Marital status**	**2,696**	**1,765**	**163**	**160**	**193**	**60**	**32**	**117**	**81**	**82**	**43**
Single	34.8	34.7	41.3	29.2	35.8	41.9	57.6	25.0	41.0	27.8	41.9
Married	60.9	62.1	53.6	61.5	60.6	43.3	39.3	68.3	46.6	69.9	58.1
**Education level^b^**	**2,770**	**1,801**	**177**	**161**	**200**	**61**	**33**	**123**	**89**	**82**	**43**
None	3.1	7.3	5.3	11.8	4.5	31.1	0	13.4	7.5	17.6	4.5
Primary	31.6	29.4	38.7	24.6	43.0	27.9	2.9	81.9	58.1	23.6	27.3
Secondary	41.6	41.4	41.3	42.0	8.5	27.9	55.8	3.1	30.1	38.6	52.7
Tertiary	23.7	21.9	14.7	21.6	44.0	13.1	38.1	1.6	4.3	20.5	15.9
**Occupation**	**1,381**	**550**	**129**	**135**	**193**	**53**	**23**	**102**	**73**	**83**	**40**
Gainful Employment	66.4	71.2	44.2	54.8	60.1	77.4	78.3	45.1	60.3	48.2	42.5
Farmer	22.3	16.4	44.2	28.1	13.5	13.2	0	50.0	27.4	9.6	2.5
Housewife	3.9	4.4	2.3	5.2	7.8	3.8	4.3	2.9	9.6	26.5	12.5
Student	3.3	4.3	3.1	8.1	17.1	3.8	4.3	2.0	2.7	4.8	10.0

Figures in bold type indicate total values.

^a^See Figure 1 for site description; ^b^Primary = 1 to 8 years of formal education, Secondary = 1 to 4 years of post-primary education, Tertiary = post-secondary, vocational or university education.

KNH, Kenyatta National Hospital.

Nearly one-third (31.6%, n = 875) had attained primary level education (up to 8 years of formal schooling), and only 4.8% (n = 133) had acquired university education. The major occupations reported included gainful employment and farming while 3.9% were unemployed (3.9%). Unemployment levels across all the sites ranged from 1.6% to 13.0%.

### Clinicians' detection rate of mental disorders

Only 114 patients (4.1%) had a mental disorder according to the clinicians' diagnoses. These included bipolar mood disorder, schizophrenia, psychosis, depression and substance abuse disorders. The file diagnoses (clinicians' detection rate) for depression ranged from none in five centres to 16.4% in Kajiado.

### Detection of mental disorders using different psychometric instruments

Table 2 shows the percentage of patients who scored positively for depression and anxiety on the BDI, NOK and LSAD.

**Table 2:** NOK, BDI and LSAD scores across all sites (% of patients)

Scores	All sites	KNH	Embu	Kiambu	Kikuyu	Kajiado	Kibera	Makindu	Naivasha	Magadi	Karuri
**BDI**	**2,563**	**1,654**	**126**	**160**	**195**	**51**	**26**	**115**	**74**	**122**	**40**
Normal	57.7	53.8	46.2	75.6	92.8	47.1	65.4	36.5	33.8	86.1	85.0
Mild	38.9	43.0	38.7	18.8	6.7	51.0	30.8	56.5	58.1	12.3	15.0
Moderate + severe	3.4	3.2	6.0	5.7	0.5	2.0	3.8	7.0	8.2	1.6	0
**NOK**	**2,348**	**1,511**	**101**	**155**	**190**	**60**	**24**	**94**	**58**	**119**	**36**
Normal	77.3	80.0	51.0	85.9	98.5	48.3	79.0	25.7	73.8	68.8	94.4
Mild	18.6	18.0	38.0	8.5	1.5	28.3	12.6	34.8	13.6	16.6	2.8
Moderate + severe	4.1	2.0	11.0	5.6	0	23.4	8.4	18.4	11.9	2.4	2.8
**LSAD:**											
Endogenous	**2,613**	**1,704**	**146**	**157**	**195**	**61**	**33**	**117**	**75**	**83**	**42**
Mild to moderate	21.4	21.0	30.8	19.7	10.8	37.7	27.3	29.9	25.3	18.1	9.5
Anxiety neurosis	**2,526**	**1,650**	**121**	**157**	**197**	**61**	**33**	**111**	**70**	**83**	**43**
Mild to moderate	11.6	9.8	19.8	8.3	1.5	37.7	15.2	37.8	20.0	6.0	7.0
General depression	**2,605**	**1,700**	**145**	**157**	**195**	**61**	**33**	**114**	**75**	**83**	**42**
Mild to moderate	26.5	27.0	35.8	19.1	13.3	36.1	24.2	39.5	30.7	25.3	9.5
General anxiety	**2,503**	**1,628**	**118**	**156**	**194**	**61**	**33**	**113**	**74**	**83**	**43**
Mild to moderate	11.5	9.3	24.5	7.7	2.0	37.7	15.1	36.3	23.0	7.2	2.3

Figures in bold type indicate total values.

BDI, Beck Depression Inventory; KNH, Kenyatta National Hospital; LSAD, Leeds Scale for the Self-Assessment of Anxiety and Depression; NOK, Ndetei-Othieno-Kathuku scale.

#### BDI

Depression was detected in patients in all the sites and the rates ranged from 7.2% to 66.2%. Overall, 42.3% of all the patients screened using the BDI had mild, moderate or severe symptoms of depression. More than half of the patients in Naivasha (66.2%), Makindu (63.5%), Embu (52.9%) and Kajiado (53.0%) had positive scores.

#### NOK

Only 1.5% of the patients in Kikuyu and 5.6% of those in Karuri screened positively for a psychiatric disorder on the NOK. Makindu (74.3%), Kajiado (51.7%) and Embu (49%) recorded high percentages of patients with positive scores.

#### LSAD

Overall, 21.4% of the patients scored positively for endogenous (severe) depression on the LSAD. General (mild) depression was recorded in 26.5% of the patients and the prevalence rates ranged from 9.5% in Karuri to 39.5% in Makindu. On average, anxiety neurosis and general anxiety were recorded in at least 11% of the patients and the levels ranged from 1.5% to 37.7% across all the centres.

#### Psychosis

Out of 85 patients who completed the psychosis questionnaire, 61% had query psychosis and 39% had frank psychosis. A diagnosis of query psychosis was made in one patient in Embu while two patients in Kibera were diagnosed with frank psychosis. However, according to their file diagnoses, psychosis was detected in only 2.9% and 0.6% of the patients in Kibera and Embu, respectively. None of the patients in Kiambu, Kikuyu, Magadi and Karuri were diagnosed with psychosis.

#### MMSE

Nearly all the patients (91.5%, n = 2,253) had normal scores on the MMSE. All the patients in Karuri (n = 44) and Kibera (n = 23) had normal scores. Only certain proportions of the patients from Makindu (52.3%, n = 86), Magadi (24.1%, n = 83), Kajiado (21.3%, n = 61) and Naivasha (15.5%, n = 84) had scores which suggested cognitive impairment.

### Comorbidity of mental disorders with hospital diagnostic categories of physical disorders (Table 3)

**Table 3:** Comorbidity of mental health disorders with diagnostic categories of physical disorders

**Categories of physical disorders**	**BDI, n (%)**	**LSAD, n (%)**				**NOK, n (%)**
		
		**Endogenous**	**Anxiety neurosis**	**General depression**	**General anxiety**	
Cancer	89 (59.6)	91 (34.1)	19 (28.6)	91 (42.2)	88 (21.6)	84 (34.5)
Cardiovascular disease	43 (16.3)	46(19.6)	45 (4.4)	46 (13.0)	44 (0)	43 (14.0)
Diabetes mellitus	157 (37.6)	162 (9.3)	155 (7.1)	151 (17.2)	151 (6.6)	141 (11.3)
Eye problems	162 (15.4)	161 (19.9)	153 (7.8)	161 (21.7)	157 (8.9)	152 (15.8)
General surgery	69 (47.8)	75 (26.7)	67 (14.9)	73 (32.9)	68 (13.2)	64 (25.0)
Peptic ulcer disease	92 (46.7)	91 (25.3)	92 (13.0)	91 (28.6)	88 (14.8)	85 (29.4)
Respiratory system	121 (41.3)	120 (28.8)	116 (9.5)	119 (26.1)	118 (11.0)	107 (24.3)
Tuberculosis	102 (41.2)	103 (34.0)	103 (22.3)	104 (38.5)	102 (19.6)	89 (37.1)
Typhoid	43 (16.3)	46 (19.6)	45 (4.4)	46 (13.0)	44 (0)	43 (14.0)
Obstetrics	226 (35.4)	232 (14.2)	233 (5.2)	250 (19.2)	250 (4.8)	224 (11.6)
Infection	124 (35.5)	123 (21.1)	126 (6.3)	123 (23.6)	125 (8.0)	118 (15.3)
Malaria	164 (28.7)	152 (19.1)	148 (16.2)	152 (23.0)	143 (13.3)	132 (32.6)
Other medical conditions	73 (37.0)	76 (23.7)	73 (11.0)	76 (28.9)	76 (10.5)	70 (78.6)
Orthopaedic/soft tissue injury	299 (44.1)	311 (23.5)	296 (9.8)	312 (32.7)	293 (10.2)	279 (28.9)
Gynaecology	155 (47.1)	157 (15.3)	151 (4.6)	154 (17.5)	154 (5.2)	149 (10.7)
HIV/AIDS	23 (52.2)	22 (31.8)	21 (28.6)	20 (30.0)	20 (30.0)	17 (64.7)
Gastric ulcer	54 (46.3)	58 (25.9)	58 (6.9)	58 (32.8)	55 (12.7)	48 (27.1)
Pain	75 (42.7)	68 (22.1)	67 (10.4)	68 (22.1)	69 (11.6)	63 (27.0)

BDI, Beck Depression Inventory; LSAD, Leeds Scale for the Self-Assessment of Anxiety and Depression; NOK, Ndetei-Othieno-Kathuku scale.

#### BDI

More than half of the patients suffering from cancer (59.6%) and HIV/AIDS (52.2%) scored for mild to moderate depression when screened using the BDI. A score of ≥ 46 (severe depression) was recorded for 30.4% of the patients with tuberculosis (TB) and 0.3% of those with orthopaedic/soft tissue injury.

#### LSAD

Between 30 and 40% of the patients suffering from cancer and HIV/AIDS had positive scores on all the depression subscales of the LSAD, whereas 20 to 30% of them scored positively on the anxiety subscales. All the patients with typhoid and cerebrovascular disease (CVD) had normal scores on the general anxiety scale.

#### NOK

Mild to severe depression detected by the NOK was recorded in 78.6% of patients with other medical conditions and 64.7% of those with HIV/AIDS.

#### Psychosis

Query psychosis was detected in two out of three general surgery patients and three out of four respiratory system patients. Frank psychosis was found with CVD (n = 1), eye problems (n = 3) and typhoid (n = 1), while all the query psychosis was found with TB (n = 2), gynaecological problems (n = 7) and HIV/AIDS. Query or frank psychosis was detected with other medical conditions (n = 3), orthopaedic/soft tissue injury (n = 5), gastric ulcer (n = 2) and pain (n = 2).

## Discussion

The highest number of respondents was recorded at the KNH and this could have been due to the fact that this is mainly a referral facility that receives patients from all over the country. The pyramid-shaped age distribution pattern of the patients in this study was similar to that of the general population. The higher number of females than males in the study was likely to be an illustration of attendance patterns, mainly at general hospitals although this finding is in contrast to the findings of a Bangladeshi study, which concluded that women appeared to have less access to public outpatient clinics than men [22]. The predominance of Christians in the sample (94.9%) was a reflection of the patterns within the general population where over 80% of Kenyans profess to be Christians [23]. The 1.4% of married subjects who were in polygamous unions and who came mainly from the predominantly rural Makindu and Kajiado was a reflection of still lingering traditional cultural practices. The low literacy rates, particularly in Kajiado where up to one-third of the subjects had received no formal schooling, could be attributed to the fact that the main economic activity here is nomadic pastoralism and the responsibility for tending livestock falls mainly on children who are supposed to be attending school. The high levels of unemployment recorded in Kibera and Karuri could be attributed to the fact that these health centres are located within the suburbs of Nairobi and are probably populated by those who could not afford to live within the city itself.

It is noteworthy that in all the facilities, the doctors detected mental illness in only 4.1% of all the patients studied, whereas instrument-assisted diagnosis yielded an average prevalence rate of 42.3% for depressive symptoms using BDI, with levels of up to 66.2% in some centres. This confirmed the notion that there is underdetection of psychiatric illnesses by doctors in medical settings [2,24]. The prevalence rate reported in this study is much higher than has been reported from studies among community members [25,26] affirming the finding that psychiatric morbidity is detected at higher levels in medical settings. The high levels of depression detected among patients in Naivasha could be attributed to urbanisation since this is a cosmopolitan setting and more people are prone to depression because of lack of traditional social support systems. High levels of depressive symptoms in Kajiado could also be attributed to traditional practices such as polygamy since women especially may have felt resentful about sharing a partner, although this study did not inquire for gender differences in depressive symptoms. Patients living in rural areas such as Kikuyu, Kiambu and Magadi were less likely to be diagnosed with depression as has been reported in other studies [27] and this finding could be attributed to the continued existence of a tightly knit society with strong family cohesion and social support systems.

Using BDI, which has been one of the most widely used instruments for screening for and diagnosing depression in general medical and surgical patients, produced higher diagnostic levels than the other instruments used in this study. This suggests that BDI could be routinely used for detecting depression in general medical facilities in Kenya, either as a screening tool for probable diagnosis of depression (for those with scores of between 12 and 42) or as a diagnostic test for depression (for those with scores above 42). However, this has the potential to create a demand that cannot be met by existing medical personnel. Nevertheless, it is better that the patients and the medical personnel know the correct diagnosis rather than subjecting patients to living with the uncertainty of their ailment. Secondly, such knowledge will provide much needed evidence-based advocacy for allocation of more resources and appropriate training of human personnel. Although less suitable, all the other instruments picked psychiatric morbidity at much higher levels than the clinicians were able to detect. All or part of the CIDI has also been used for general screening in various settings [21].

Only 85 out of 2,770 (3.1%) subjects had either query or frank psychosis and this finding was similar to what was found in another study although the latter study was conducted among the general population [28]. This level may have been an illustration of the true picture or an indication that the prevalence of psychosis in general hospitals is low since it is expected that such patients should be admitted in psychiatric hospitals. However, it should be noted that psychosis was one of the disorders that had been recognised by non-psychiatric clinicians since probably because of their very nature and compared to depressive symptoms, psychotic symptoms are relatively simple to detect.

Comorbidity of psychiatric disorders with specific physical disorders was noted in this study. The highest comorbidity rates were recorded with HIV/AIDS, TB, CVD, cancer, gynaecological and genitourinary conditions. This high level of mental disorders could be related to the chronicity of these conditions. Other studies have made similar observations [7-9] and one study has more specifically demonstrated that there are high levels of depression among HIV-infected individuals [29].

Despite wide variations in the prevalence of mental disorders in different facilities, the overall pattern of a high level of mental disorders detected with greater frequency in inpatients than in outpatients was similar from primary level to the tertiary level of health care. Another finding common to all facilities was that most of these disorders remained undiagnosed by clinicians. It was significant that at the higher levels of health provision, less mental disorders were recognised. It was likely that as medical personnel became more specialised in their field, they were less likely to make any other consideration. At the KNH (a general referral facility), Makanyengo [30] found that only 8.7% of the patients from the wards were referred, which constituted 9.6% of all the referrals to the psychiatric services.

These findings have several policy and practice implications. There is need for an increased awareness of the prevalence of psychiatric symptoms in patients attending general medical facilities at all levels, and particularly in those already admitted for one or more physical conditions. This calls for sensitisation at all levels of medical education, from undergraduate to postgraduate level. For those already in service, there is need for continuing medical education (CME) on mental health. Thirdly, there is need for routine use of screening instruments to assist in making diagnoses. The importance of involving medical professionals at all levels is seen in the fact that even in the foreseeable future, Kenya like most African countries will not have sufficient psychiatrists to provide these services [15].

This study had limitations. There were varied response rates for all the variables across all the sites since not all the patients completed all the questionnaires. This meant that comparison of the results across the sites could only be made cautiously. The use of self-administered instruments and scales aimed for symptom measurement may have led to diagnostic overestimation. Furthermore, the use of several instruments produced different detection levels of psychiatric morbidity, especially for depression and anxiety. However, this served to suggest that BDI, for which there is more data worldwide on use in similar circumstances, could be the most suitable for routine use.

Although attempts were made to stratify and then sample systematically within each stratum, there is some likelihood that the samples were not completely representative. Even with this limitation, this study provides credible evidence to initiate appropriate policies and practices to address mental health in general primary and hospital facilities and provides strong evidence for liaison psychiatry with general medical facilities.

## Conclusion

There is high prevalence of psychiatric morbidity in Kenyan general medical facilities but this largely goes undiagnosed and therefore, unmanaged. The more specialised medical facilities get in the various general and surgical disciplines, the less recognised mental disorders become. Chronic conditions had the highest comorbidity with mental disorders, particularly depression and anxiety. These findings call for continuing education on mental health at all levels of general and surgical facilities, and also for routine screening for mental disorders.

## Competing interests

The authors declare that they have no competing interests.

## Authors' contributions

DMN contributed to conception and design of the study and was involved in drafting the manuscript and revising it critically for intellectual content. LIK participated in acquisition, analysis and interpretation of data and was involved in drafting the manuscript and revising it critically for intellectual content. MWK contributed in acquisition of data and was involved in interpretation of data. VNM participated in acquisition, analysis and interpretation of data and was involved in drafting the manuscript. FAO-O participated in acquisition of data and was involved in drafting the manuscript. DAK was involved in acquisition of data and assisted in interpretation of data. All the authors have read and approved the final manuscript.
